# Letter regarding “Anteriolateral versus anterior–posterior electrodes in external cardioversion of atrial fibrillation: A systematic review and meta‐analysis of clinical trials”

**DOI:** 10.1002/clc.24249

**Published:** 2024-03-04

**Authors:** Stephanie T. Nguyen, Emilie P. Belley‐Côté, William F. McIntyre

**Affiliations:** ^1^ Department of Medicine McMaster University Hamilton Ontario Canada; ^2^ Population Health Research Institute Hamilton Ontario Canada

To the Editor,

The optimal electrode pad placement for successful cardioversion of atrial fibrillation (AF) remains unknown. In a systematic review and meta‐analysis of 11 trials, Motawea et al. concluded that anterolateral (AL) positioned pads are more effective than anterior‐posterior (AP) positioned pads for electrical cardioversion of patients with AF (odds ratio: 1.40, 95% confidence interval [CI]: 1.02–1.92, *p* = .04).^1^

We previously reported a systematic review and meta‐analysis of randomized controlled trials of techniques to improve cardioversion success. In contrast to Motawea et al., we found that overall cardioversion success did not differ when comparing AL to AP‐positioned pads (Risk ratio: 1.01, 95% CI: 0.96–1.06, *p* = .70).^2^

Two principal issues drive the differences between our studies' results. The first issue relates to study selection. Motawea et al. omitted four randomized trials that were included in our meta‐analysis, this represents 389 extra participants or 21% more participants.^3^, ^4^, ^5^, ^6^ They also erroneously included one prospective observational cohort study (111 participants).^7^ The second issue relates to data appraisal and/or data abstraction. The authors have recorded incorrect values for the trials by Alp et al. and Botto et al.^8^, ^9^ For these two trials, the authors appear not to have followed the intention to treat principle; some abstracted values represent cross‐overs rather than the pad placements to which the patients were initially randomized.

We, therefore, advise caution when interpreting the study by Motawea et al.; AL pad placement has not been shown to be superior to AP placement. A definitive trial addressing the question is ongoing (NCT05511389).^10^ An additional important limitation of these data not raised by Motawea et al., is that pad placement has rarely been tested in studies where participants were consistently receiving other co‐interventions that have been proven effective (i.e., high energy and biphasic shocks). A large randomized controlled trial comparing pad placement in patients with AF with other best practices in place is thus warranted.

## CONFLICT OF INTEREST STATEMENT

The authors declare no conflict of interest.
